# Lung function changes and complications after lobectomy for lung cancer in septuagenarians

**DOI:** 10.4103/1817-1737.49413

**Published:** 2009

**Authors:** Dragan Subotic, Dragan Mandaric, Gordana Radosavljevic, Jelena Stojsic, Milan Gajic

**Affiliations:** *Institute for Lung Diseases, Clinical Center of Serbia, Belgrade, Serbia*; 1 *Institute for Medical Statistics, Faculty of Medicine, Belgrade, Serbia*

**Keywords:** Lobectomy, lung cancer, pulmonary function

## Abstract

**BACKGROUND:**

In septuagenarians, lobectomy is the preferable operation, with lower morbidity than for pneumonectomy. However, the 1-year impact of lobectomy on lung function has not been well studied in elderly patients.

**MATERIALS AND METHODS:**

Retrospective study including 30 patients 70 years or older (study group), 25 patients with chronic obstructive pulmonary disease (COPD) under 70 years (control group 1), and 22 patients under 70 years with normal lung function (control group 2) operated for lung cancer in a 2-year period. The study and control groups were compared related to lung function changes after lobectomy, operative morbidity, and mortality.

**RESULTS:**

Postoperative lung function changes in the elderly followed the similar trend as in patients with COPD. There were no significant differences between these two groups related to changes in forced expiratory volume in the first second (FEV_1_) and vital capacity (VC). Unlike that, the pattern of the lung function changes in the elderly was significantly different compared with patients with normal lung function. The mean postoperative decrease in FEV_1_ was 14.16% in the elderly, compared with a 29.23% decrease in patients with normal lung function (*P* < 0.05). In the study and control groups, no patients died within the first 30 postoperative days. The operative morbidity in the elderly group was significantly lower than in patients with COPD (23.3% vs. 60%).

**CONCLUSIONS:**

The lung function changes after lobectomy in the elderly are similar to those in patients with COPD. The explanation for such a finding needs further investigation. Despite a high proportion of concomitant diseases, the age itself does not carry a prohibitively high risk of operative mortality and morbidity.

Lung resection for lung cancer in patients in the age group of 70 years or older still represents a surgical challenge, both from the surgical and the oncological standpoint. The peak incidence age for lung cancer occurrence was around 60 years in 1987, but has now shifted to the age of 70–74 years. In addition, there is a general trend worldwide of an increasing incidence of lung cancer in the elderly.[1]

The rapidly aging population in these countries led to the increase in the number of elderly patients with lung cancer, making it the second-leading cause of cancer death among this age group.[2]

Despite a widespread belief that advanced age excludes surgery by itself, recent studies have shown that lung cancer resection can be justified in these patients, but only if properly selected.[3] Of late, the age limit for surgery has even been pushed upwards to 80 years.[4]

As pneumonectomy in the elderly definitely carries a significantly higher operative risk compared with younger patients, a broad consensus exists that lobectomy is the preferable operative procedure, rendering better survival than for conservative resection, with lower morbidity than for pneumonectomy. However, the 1-year impact of lobectomy on lung function has not been well studied in elderly patients.[5]

Having this in mind, together with data that advanced age and chronic obstructive pulmonary disease (COPD)[6] have an adverse effect on operative mortality, we intended to analyze the pattern of lung function change after lobectomy in the elderly and to compare it with a group of COPD and with a group of younger non-COPD patients. Also, the aim of the study was to analyze and discuss causes of eventual differences in operative mortality and morbidity between these groups.

## Materials and Methods

Retrospective study including 30 patients 70 years or older (study group), 25 patients with COPD under 70 years (control group 1), and 22 patients under 70 years with normal lung function (control group 2), who underwent a lobectomy for primary lung cancer in a 2-year period (2004 and 2005).

The structure of the study and control groups is presented in Table 1.

**Table 1:** Structure of the study and control groups

Group	*N*	Age interval/mean	M:F	RUL	RLL	Bil	ML	LUL	LLL
Study	30	70–76/72.2	3:28	9	7	2	-	6	6
Control 1	25	45–69/58.9	4:1	10	6	-	1	4	4
Control 2	22	38–68/56.7	6:3	5	4	-	1	7*	5

M-male; F-female; RUL-right upper lobectomy; RLL-right lower lobectomy; Bil-bilobectomy; ML-middle lobe resection; LUL-left upper lobectomy; LLL-left lower lobectomy

* In one patient-LUL + chest wall resection

Postoperative patohistology revealed that no lymph node metastases existed in 18 patients (12T2N0, 4T1N0, 2T3N0), metastases in the hilar/interlobar lymph nodes existed in 10 patients (8T2N1, 1T1N1, 1T3N1), while only two patients had metastases in the mediastinal lymph nodes (2T2N2). Squamous cell and adenocarcinoma existed in 26 and four patients, respectively.

### Pre-operative work up

In the study group, pre-operative work up related to lung cancer was the same as in the control groups (standard clinical and laboratory investigations, bronchoscopy, high-resolution computed tomography of the thorax and upper abdomen, and respiratory function tests).

In the control group 1, combined bronchodilator therapy was applied. Patients with forced expiratory volume in the first second (FEV_1_) and 100 FEV_1_/vital capacity (VC) greater than 60% at control spirometry were referred directly to surgery. Patients with FEV_1_ and 100 FEV_1_/VC lower than 60% at control spirometry were subjected to perfusion scintigraphy of the lungs in order to calculate the predicted postoperative FEV_1_ (ppoFEV_1_).

In the control group 2, consisting of younger aged patients with nonimpaired lung function, no additional work up (outside the previously described related to the local and distant tumor spread) was carried out.

### Data analysis

The obtained demographic and clinical data, including age, gender, pulmonary function, comorbidity, quality of life after the operation, as well as peri-operative data, consisting of surgical procedure, pathologic stage, and operative morbidity and mortality, were entered into the database.

In the study and control groups, pre-operative absolute and percent values of FEV_1_, VC, forced expiratory flows at 50% and 25% vital capacity (FEF_50_ and FEF_25_), and 100 FEV_1_/VC were compared with corresponding postoperative values, obtained at least 3–6 months after the operation. The differences between pre-operative and postoperative values of FEV_1_, VC, FEF_50_, and FEF_25_ were calculated and compared between the groups.

The study and control groups were compared related to survival. Survival was estimated by the Kaplan–Meier method.

### Statistics

Statistical significance of differences between the groups related to pre-operative lung function parameters was assessed by using a one-way ANOVA test. Statistical analysis of differences in lung function changes after the operation was performed using a Kruscal–Wallis test and Mann–Whitney *U*-test. Differences between the groups related to the existing comorbidity were assessed using a χ^2^ test. Within the same analysis, differences with regards to the existence of one or more than one comorbidities were assessed using a Fisher's exact test.

## Results

### Postoperative lung function changes

In the study group, in 13/30 (43.3%) patients, the 100 FEV_1_/forced vital capacity (FVC) was <70%, representing the existence of COPD according to the GOLD criteria. Pre-operative baseline values of the lung function parameters in the analyzed groups are presented in Table 2.

**Table 2:** Pre-operative lung function parameters in the analyzed groups

		Median	Minimal	Maximal
Elderly	FEV_1_	2640	1100	3950
	VC	3845	2020	5010
	FEF_50_	52	13.4	131
	FEF_25_	39.8	14	109.8
Limited lung function	FEV_1_	1640	950	2420
	VC	3090	1540	4420
	FEF_50_	22	8.5	45.7
	FEF_25_	23.5	9.1	59.4
Normal lung function	FEV_1_	2640	2230	3800
	VC	3840	3220	5740
	FEF_50_	62.5	38	109
	FEF_25_	59	31	94
*P* (e:l)	FEV_1_:<0.001;	VC:<0.05;	FEF_50_:<0.001;	FEF_25_:<0.05
*P* (e:n)	FEV_1_:<0.05;	VC:<0.05;	FEF_50_:>0.05;	FEF_25_:<0.05
*P* (l:n)	FEV_1_:<0.001;	VC:<0.01;	FEF_50_:<0.001;	FEF_25_:<0.001

FEV_1_ - forced expiratory volume in the first second; VC-vital capacity; FEF_50_ - forced expiratory flow at 50% VC; FEF_25_ - forced expiratory flow at 25% VC; *P*-statistical significance between the patient groups in relation to a particular parameter (FEV_1_, VC, FEF_50_, FEF_25_) (95% confidence interval); *P* (e:l)-elderly vs. patients with limited lung function; *P* (e:n)-elderly vs. patients with normal lung function; *P* (l:n)-patients with limited vs. patients with normal lung function

Comparison between pre-operative and postoperative lung function in the study and control groups is presented in Table 3.

**Table 3:** Postoperative lung function changes

		Median	Minimal	Maximal
Elderly	ΔFEV_1_	14.16	−18.26	59.55
	ΔVC	12.77	−4.60	52.48
	ΔFEF_50_	24.29	−101.79	65.91
	ΔFEF_25_	−1.06	−133.5	100
Limited lung function	ΔFEV_1_	6.23	−39.6	40.18
	ΔVC	12.57	−47.4	40.19
	ΔFEF_50_	−0.66	−242.86	41.56
	ΔFEF_25_	−7.58	−207.69	49.20
Normal lung function	ΔFEV_1_	29.23	3.83	71.84
	ΔVC	27.66	5.14	60.36
	FEF_50_	37.72	−50.77	79.10
	ΔFEF_25_	25.35	−62.5	73.53
*P* (e:l)	FEV_1_:>0.05;	VC:>0.05;	FEF_50_:<0.05;	FEF_25_:>0.05
*P* (e:n)	FEV_1_:<0.05;	VC:<0.05;	FEF_50_:>0.05;	FEF_25_:>0.05
*P* (l:n)	FEV_1_:<0.05;	VC:<0.05;	FEF_50_: 0.001;	FEF_25_:<0.05

ΔFEV_1_-percent difference between FEV_1_ (ml) before vs. FEV_1_ (ml) after lobectomy; ΔVC-percent difference between VC (ml) before vs. VC (ml) after lobectomy; ΔFEF_50_-percent difference between FEF_50_ (%) before vs. FEF_50_ (%) after lobectomy; ΔFEF_25_-percent difference between FEF_25_ (%) before vs. FEF_25_ (%) after lobectomy; *P*-statistical signficance between the patient groups in relation to a particular parameter (ΔFEV_1_, ΔVC, ΔFEF_50_, ΔFEF_25_) – asymp. sig. (2-tailed); *P* (e:l)-elderly vs. patients with limited lung function; *P* (e:n)-elderly vs. patients with normal lung function; *P* (l:n)-patients with limited vs. patients with normal lung function

Postoperative lung function changes in the elderly followed the same trend as in patients with COPD. Although the postoperative loss in FEV_1_ was clearly lower in the COPD group, there were no significant differences between these two groups related to FEV_1_ and VC.

Unlike that, the pattern of the lung function changes in the elderly was significantly different compared with patients with normal lung function. The median postoperative decrease in FEV_1_, expressed as percent loss related to pre-operative values, was 14.16% in the elderly, compared with a 29.23% decrease in patients with normal lung function (*P* < 0.05). The postoperative loss in VC was also significantly lower in the elderly than in patients with normal lung function (12.77%, 27.66%; *P* < 0.05). Although the loss in the small airways function (FEF_50_ and FEF_25_) was evidently lower in the elderly, these differences did not reach the level of statistical significance.

### Operative mortality and morbidity

Comorbidity in the study and control group 1 is presented in Table 4. In the control group 2, there was no major comorbidity. In the study and control group 1, no comorbidity existed in 23.35% and 44% of the patients respectively. While in the study group more than one associated disease existed in 11/23 (47.82%) of the patients with comorbidity, it was registered in only 2/14 (14.28%) of the patients with comorbidity in the COPD group.

**Table 4:** Comorbidity in the study group and control group 1

	Elderly		COPD		*P*
			
	*n*	%	*n*	%	
Without comorbidity	7/30	23.35	11/25	44	>0.05
With comorbidity	23/30	76.65	14/25	56	
>One associated diseases	11/23	47.82	2/14	14.28	<0.05*
One single associated disease	12/23	52.18	12/14	85.72	
Arterial hypertension	15/23	65.2	3/14	21.42	<0.05
Arrhythmia	7/23	30.4	5/14	35.71	>0.05
Myocardiopathy	4	17.39	1/14	7.14	>0.05
Angina pectoris	3	13	-	-	
Myocardial infarction	1	4.34	-	-	
Chronic renal insufficiency	1	4.34	-	-	
Diabetes	-	-	2/14	14.28	
Anemia	-	-	2/14	14.28	
Other	3	13	4/14	28.56	

*P*-statistical significance between the patient groups in relation to particular analyzed parameters (χ^2^ test)

* Fisher's exact test

In the study and control groups, no patients died within the first 30 postoperative days. In the elderly group, postoperative complications occurred in 7/30 (23.3%) of the patients (prolonged air leak in six and partial wound dehiscence in one patient).

In the COPD goup, postoperative complications occured in 15/25 (60%) of the patients. One single complication occurred in 14 patients while only one patient had more than one complication. Prolonged air leak was registered in 10 patients as the only complication. Pulmonary embolism, respiratory insufficiency, pneumothorax, and gastric ulcer occurred in an additional four patients as a single complications while one patient experienced prolonged air leak, arrhythmia, and partial wound dehiscence.

In the control group 2, operative morbidity was 31.81%. Prolonged air leak and arrhythmia occurred in 4/22 (18.2%) and in 3/22 (13.6%) of the patients, respectively.

The obtained differences in the operative morbidity between the elderly and the COPD group are statistically significant (χ^2^ 7.639; *P* < 0.001). The lower operative morbidity in the elderly compared with the control group 2 is not statistically significant (χ^2^ 0.464; *P* > 0.05).

### Survival

**Figure 1:**
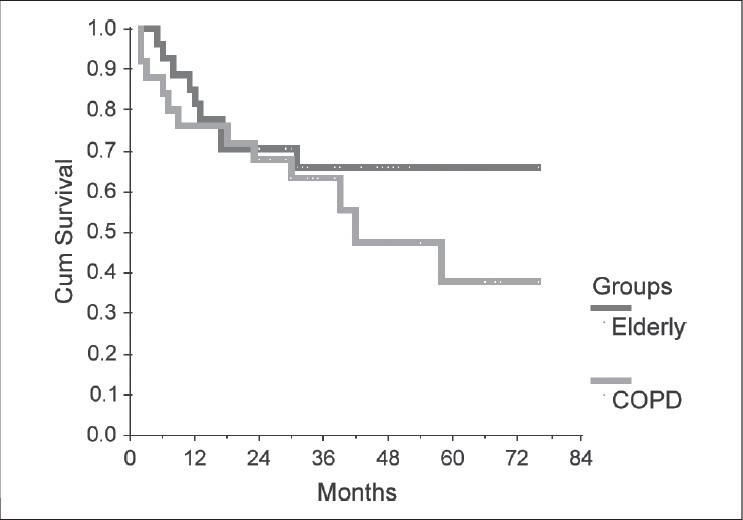
Survival is months: Eldery vs.COPD

The overall survival of elderly and COPD patients is presented in Figure 1. Five-year survival in the elderly and the COPD group was 65.9% and 37.9%, respectively (median survival 76 ± 58 vs. 42 ± 13.3 months). Although evident, the survival difference was not statistically significant.

## Discussion

### Incidence, patients' characteristics, selection criteria

Two facts should be pointed out when discussing lung resection for lung cancer in the elderly: First, the common practice of offering less-aggressive treatment to this subgroup of patients, including a nonoperative approach or less than an anatomic resection, led to the situation that patients ≥65 years old, with localized lung cancer, were only one-third as likely to undergo resection compared with younger patients. Indeed, for each decade of life after 65 years of age, the likelihood of undergoing resection declined by 65%[7]; second, the previously mentioned increase in the life expectancy for octogenarians (8.6 years in the US) translates into an overall 5-year survival of 80% for an age- and gender-matched population. As the majority of this time is anticipated to be years of active and independent life, it becomes clear that an octogenarian's survival and quality of life will be influenced mostly by their cancer-related mortality rather than by their age.[8]

Related to these facts, the elderly group in the present study accounts for 7.15% of a total number of 419 patients with lobectomy (389) and bilobectomy (30) in the analyzed period. This proportion corresponds to that in rare surgical series containing these data.[9]

Our study confirmed that 26/30 (86.6%) of the patients who were alive during the 2-year follow-up period were capable of active and good quality life, including regular visits to the outpatient clinic.

In the study group, the M:F ratio was 3.28. Although this ratio varies between 1.6 and 11 according to the literature, in all studies, the male gender is significantly higher in the elderly compared with younger patients. In some studies, in the younger group, the proportion of women is twice as high as in the older group.[10]

In our study, although the mean age in control groups was younger (58.9 and 56.7 years) than in the study group, the M:F ratio did not follow the aforementioned pattern, probably because those were not true “younger” groups. Even among seven patients aged 39–45 years, we did not confirm a recently reported pattern of decrease in the M:F ratio from 1.62 to 0.89 in the group <40 years old.[11]

As already mentioned, calculation of the predicted ppoFEV_1_ was mandatory because it was shown long ago that an estimated ppoFEV_1_ value under 43% correlated with the need for home oxygen and that the probability of survival in older patients with low calculated ppoFEV1% was only 17%.[12] Although the association of low predicted pulmonary capacity for carbon monoxide diffusion (DLco), not only with the need for hospitalizations and home oxygen but also with operative mortality[13] was clearly shown, in our series, DLco was not routinely carried out, except in a few patients with mild to moderate obstructive ventilatory disorders. Arterial blood gas analyses were mandatory in our group of the elderly, although rare studies demonstrated that arterial blood gases alone were not a predictor of postoperative complications.[14]

Related to the staging status in the elderly group, predominance of T1/T2 and N0/N1 tumors does not reflect the true incidence of these tumors in the overall elderly population because the patient selection was limited to patients with lobectomy. For the same reason, we did not compare the staging status between the study and the control groups. Such a comparison would nevertheless make sense in the absence of the aforementioned limitations because some recent studies showed no statistical difference between younger and older cancer patients, contrary to some studies performed 10 years ago in which young patients had a relatively delayed diagnosis and more advanced disease.[15] This could be due to the slow progress of lung cancer in the elderly or to the tendency of younger patients to ignore or misinterpret some changes in their health. The predominance of the squamous cell carcinoma (86.6% patients) in the present study confirms previous reports that found this tumor type as the most frequent in the elderly.

### Postoperative lung function changes

The reason for specifically addressing the pattern of lung function change after lobectomy in this study was as follows: The well-known early functional deficit both in FEV_1_ and FVC after lobectomy, followed by later functional recovery at 6 months, was based on studies that included patients with an average age of only 64.2 years.[16] The mean age of 72.2 years (70–76) in our group gave the opportunity to analyze these functional changes, still not fully elucidated, but in an older group of patients.

We recently demonstrated smaller loss in FEV_1_ and improved function of the small airways in patients with COPD compared with patients with normal lung function,[17] a finding that was consistent with recent reports.[18] But, the similar trend in the postoperative function changes in the elderly and in patients with COPD, as demonstrated in the present study, is not easy to be explained. It could be only in part due to the proportion of patients with COPD in the elderly group – 13/30 (43.3%). This percentage of COPD patients could influence the pattern of the lung function change by approaching it to that in COPD patients, but in 12/13 patients with obstructive ventilatory disorders in the elderly group, slight (4 pts) or moderate (8 pts), while in only one patient severe COPD existed. Unlike this, 14 (56%) of the patients in the control group of patients with COPD had moderate or severe obstructive ventilatory disorders.

Most of the literature data refer to differences in postoperative lung function changes only between the elderly and the young patients, not between the elderly and the younger COPD patients. Some of these studies demonstrated the significantly higher percentage of patients with VC <70% and PaO_2_ <70mmHg in the elderly vs. younger patients, associated with smaller decrease in both VC and FEV_1_ in younger patients.[5,15]

### Operative morbidity and mortality

In the study group, three-fourth of the patients had concomitant diseases, compared with 56% of such patients in the group with COPD. The main difference between these groups related to comorbidity was a significantly higher number of patients with more than one associated disease in the group of elderly – nearly half of the patients vs. only 2/14 patients in the COPD group. Although the percentage of patients with comorbidity was slightly higher in our group than in rare studies containing these data, the distribution of diseases was similar, with arterial hypertension and cardiac arrhythmias being dominant.[19]

When discussing 0% operative mortality in the present study, it should kept in mind that the time trend in operative mortality in the elderly decreased, from ∼20% in the initial reports through still high 13% in some series to 3–7% in some recent reports.[20,21] It is also of importance that the proportion of pneumonectomies varied in these series, from 51% in the series of Thomas through 19% in the series of Ginsberg to 10% of Breyer's[20] and 6% of Ishida's series.[21] Although this proportion certainly influences the mortality rates, it would be a misleading conclusion that only patients with pneumonectomy and octogenarians are at high risk of early postoperative death. In 1992, Romano and Mark demonstrated that the adjusted odds ratio for death was 3.6 times greater in septuagenarians when compared with patients younger than age 60.[22] In the aforementioned series of Thomas (10) with 7.4% in-hospital deaths in the elderly, mortality rates following bilobectomy, lobectomy, and lesser resection were 4/34 (11.11%), 22/291 (7.56%), and 0/39, respectively. Among factors that could contribute to this still high mortality rate after lobectomy in the elderly, lateral position and one-lung ventilation with alternative lung inflation and deflation during a long period of surgery could influence the physiological status of the lung in the elderly.[23]

While the early-stage lung cancer in the octogenarian is a fatal disease, in recent studies, the long-term survival of septuagenarian patients has been shown to be comparable to that of younger patients.[24] It means that early postoperative deaths can be prevented in different ways. In the analyzed group of elderly patients, bronchoscopic aspiration of accumulated bronchial secretions with imminent or developed athelectasis was preferred to intensive physiotherapy alone. In addition, early rising from bed was the common policy, both in our and also in patients from other reported series.[25]

The cause of the lower complication rate (23.3%) in the elderly group of the present study compared with the 38–55% complication rate most frequently cited in the literature is the consequence of two facts: first, we did not count as complications pre-existing diseases, presenting as comorbidity. Second, in most published reports, the complication rates referred to all types of resections, including standard and extended pneumonectomies. The lower complication rate in the elderly compared with the COPD patients in the present study, despite the opposite comorbidity rate in these groups, can be explained by the poor quality of lungs in COPD patients, which accounted for most postoperative complications in this group (10/15 patients had prolonged air leak as the only complication).

However, operation-adjusted data demonstrated a 22.8% cardiovascular and a 14.4% nonfatal pulmonary complication rate after resections lesser than pneumonectomy in the elderly. Differences in the operative morbidity in the present study between the elderly and the COPD group (23.3% vs. 60%) and the diversity of complications in the latter, even with 44% COPD patients without comorbidity, confirmed previous reports that advanced age should not be regarded as an independent prognostic factor if resection lesser than pneumonectomy is anticipated.

### Survival

Survival of the elderly patients in the present study was slightly superior than in COPD patients. But, when discussing survival in the elderly patients, it is likely that septuagenarians should be analyzed separately from octogenarians. Otherwise, the overall pool of data contributes to confusion, ranging from better survival in younger age groups (despite more advanced-staged disease) to completely comparable survival.[26–28] Moreover, while some authors consider early-stage lung cancer in the octogenarian a fatal disease, other reports showed similar survival figures between patients aged 70 and 74, 75 and 79, and beyond 80 years.[29–31] It should be kept in mind that the proportion of stage I patients in series reporting elderly patients is higher than in series also reporting patients from all age groups. Thus, our data are in line with reports finding similar overall 5-year survival in elderly and younger patients,[32] provided the expected survival benefit is counterweighted with appropriately assessed operative risk.

In conclusion, the pattern of the lung function changes after lobectomy in the elderly is similar to that in patients with COPD. The explanation for such a finding needs further investigation. Despite a high proportion of concomitant diseases, the age by itself does not carry a prohibitively high risk of operative mortality and morbidity.
